# C-954-09. Improving Burn Rehabilitation Therapy Education and Competency Through Utilization of a Burn Rehab Collaborative

**DOI:** 10.1093/jbcr/irag033.141

**Published:** 2026-04-06

**Authors:** B Gina Chrysler

**Affiliations:** Eastern Idaho Regional Medical Center, Idaho Falls, ID

## Abstract

**Introduction:**

The American Burn Association (ABA) has standards of clinical competency for burn rehabilitation therapists to meet best practice standards. Burn Rehabilitation Therapists Competency Tool (BRTCT-Version 2) is utilized by occupational and physical therapists in burn centers to define the skills and knowledge needed for hospitalization, inpatient/outpatient rehabilitation, and long-term rehabilitation. At a newer burn center, it is a challenge to get effective education for burn rehabilitation therapists, since there are not many continuing education methods available to therapists new to this area of practice. To assist with meeting standards set in BRTCT-Version 2 and support the education and growth of all burn therapists in our healthcare system, a Burn Rehab Collaborative was developed.

**Methods:**

The Burn Rehab Collaborative was developed with the use of a telecommunication meeting platform. These meetings involve teams of therapists with burn rehabilitation experience ranging from 20 years to less than 1 year. BRTCT-Version 2 was utilized to guide and drive topics of education and collaboration throughout the teams.

To determine the efficacy of the Burn Rehab Collaborative, a survey was carried out both before and after the first meeting with the following meetings only having a preceding survey. The survey queried the therapists for their knowledge and experience on the topics from the previous meeting as well as the topics for the following meeting.

**Results:**

Over the past 5 months, the collaborative has covered topics specific to burn rehabilitation including splinting, scar management, positioning, and application of cutaneous functional units.

On average, 16 therapists respond to the surveys with experience on topics of discussion ranging from 5% reporting no experience, 40% reporting minimum, 33% reporting moderate, and 22% reporting extensive. Over 85% of the therapists reported learning something new from the previous collaborative meeting with 60% reporting that they had applied a new technique that was learned in the meeting.

**Conclusions:**

The Burn Rehab Collaborative promotes collaboration and sharing between rehabilitation teams across our healthcare system. It has improved the confidence and knowledge of burn rehabilitation therapists on application of techniques and practices. It has also created a platform for sharing solutions to challenging conditions and barriers that the rehabilitation teams are experiencing with current and past burn patient cases.

**Applicability of Research to Practice:**

The use of the Rehab Burn Collaborative is an effective way to share knowledge and experience between therapists as well as build understanding and confidence to improve the outcomes of burn rehabilitation patients. Focusing on different sections of the BRTCT-Version 2 ensures that teams are gaining knowledge in areas of competency required by the ABA.

**Funding for the Study:**

N/A.

